# Sex differences in distribution and identity of aromatase gene expressing cells in the young adult rat brain

**DOI:** 10.1186/s13293-023-00541-8

**Published:** 2023-09-01

**Authors:** Jana Immenschuh, Stefan Bernhard Thalhammer, Inger Sundström-Poromaa, Anat Biegon, Sylvie Dumas, Erika Comasco

**Affiliations:** 1 Department of Women’s and Children’s Health, Science for Life Laboratory, Uppsala University, Uppsala, Sweden; 2 Department of Women’s and Children’s Health, Uppsala University, Uppsala, Sweden; 3 Department of Radiology and Neurology, Stony Brook University School of Medicine, Stony Brook, NY USA; 4 Oramacell, Paris, France

**Keywords:** Aromatase, Brain, Cyp19a1, Estrogen, Expression, FISH, Gene, mRNA, Rat, Sex differences

## Abstract

**Background:**

Aromatase catalyzes the synthesis of estrogens from androgens. Knowledge on its regional expression in the brain is of relevance to the behavioral implications of these hormones that might be linked to sex differences in mental health. The present study investigated the distribution of cells expressing the aromatase coding gene (*Cyp19a1)* in limbic regions of young adult rats of both sexes, and characterized the cell types expressing this gene.

**Methods:**

*Cyp19a1* mRNA was mapped using fluorescent in situ hybridization (FISH). Co-expression with specific cell markers was assessed with double FISH; glutamatergic, gamma-aminobutyric acid (GABA)-ergic, glial, monoaminergic, as well as interneuron markers were tested. Automated quantification of the cells expressing the different genes was performed using CellProfiler. Sex differences in the number of cells expressing *Cyp19a1* was tested non-parametrically, with the effect size indicated by the rank-biserial correlation. FDR correction for multiple testing was applied.

**Results:**

In the male brain, the highest percentage of *Cyp19a1*^+^ cells was found in the medial amygdaloid nucleus and the bed nucleus of stria terminalis, followed by the medial preoptic area, the CA2/3 fields of the hippocampus, the cortical amygdaloid nucleus and the amygdalo-hippocampal area. A lower percentage was detected in the caudate putamen, the nucleus accumbens, and the ventromedial hypothalamus. In females, the distribution of *Cyp19a1*^+^ cells was similar but at a lower percentage. In most regions, the majority of *Cyp19a1*^+^ cells were GABAergic, except for in the cortical-like regions of the amygdala where most were glutamatergic. A smaller fraction of cells co-expressed *Slc1a3*, suggesting expression of *Cyp19a1* in astrocytes; monoaminergic markers were not co-expressed. Moreover, sex differences were detected regarding the identity of *Cyp19a1*^+^ cells.

**Conclusions:**

Females show overall a lower number of cells expressing *Cyp19a1* in the limbic brain. In both sexes, aromatase is expressed in a region-specific manner in GABAergic and glutamatergic neurons. These findings call for investigations of the relevance of sex-specific and region-dependent expression of *Cyp19a1* in the limbic brain to sex differences in behavior and mental health.

**Supplementary Information:**

The online version contains supplementary material available at 10.1186/s13293-023-00541-8.

## Plain language summary

It is known that there are differences in the way males and females are mentally affected. These have been in part attributed to the effect of sex hormones, such as estrogen and testosterone. Within the framework of sex-specific medicine, it is therefore important to understand the biological substrates of sex-specific systems in the brain that are involved in any of these differences. The present study investigated the enzyme responsible for the synthesis of estrogen in the brain, to identify where it is expressed in the brain and to characterize the cells in which it is expressed. To this end, female and male young adult rats were studied. Brain slices including regions of relevance to, among others, emotion processing, were analyzed using fluorescent probes for the genes of interest and visualized using microscopy. Automated cell counting illustrated sex differences, with males displaying greater expression of the aromatase gene, compared with females, in several regions. The aromatase gene was expressed together with genes for the major inhibitory and excitatory neurotransmitters.

**Supplementary Information:**

The online version contains supplementary material available at 10.1186/s13293-023-00541-8.

## Highlights

- Aromatase gene expression is higher in the limbic brain of males.
- GABAergic and glutamatergic cells express the aromatase gene region-specifically.
- Astrocytes also show expression of the aromatase gene.
- Sex differences were even found in co-expression patterns.

**Supplementary Information:**

The online version contains supplementary material available at 10.1186/s13293-023-00541-8.

## Introduction

Many studies on both the human and the animal brain predominantly investigated male subjects, which can lead to oversimplification and one-sided view, as growing evidence suggests that knowledge on male brains is not universally applicable to females [1]. Sex differences in brain morphology and function as well as neurochemistry have been reported and might be linked to sex differences in the incidence and/or nature of psychiatric disorders [1]. Profound effects of gonadal hormones on the organizational make-up of the brain during development are known, and besides exerting a regulatory role in reproductive functions, they also influence brain function during adulthood [2, 3]. Thus, gonadal hormones such as androgens and estrogens are likely to play a role in emotional and cognitive functions; however, the present knowledge about the underlying biology is scarce [4, 5].

Aromatase, which is encoded by the gene *Cyp19a1* (cytochrome P450 family 19 subfamily A member 1), catalyzes the conversion of the androgens androstenedione and testosterone, into the estrogens estrone and 17β-estradiol, respectively. Therefore, it is able to acutely and chronically control the androgen–estrogen ratio in the brain [6]. In addition to functions related to reproduction, aromatase plays a role in neural proliferation and neuroprotection, as well as in pathological processes [7]. Notably, in rodents, aromatase has been implicated in behavioral functions, including sexual behavior and aggression, and is suggested to play a role in cognition and memory [2, 7–9].

Despite the potential impact of aromatase on several brain functions, mapping and characterization of *CYP19A1/Cyp19a1*-expressing cells is limited, both in humans and rodents, respectively, and often lacks investigation of sex differences. In rodents, studies on *Cyp19a1* mRNA expression throughout the rat brain have indicated that the highest levels of *Cyp19a1* mRNA are found in the amygdala, the bed nucleus of the stria terminalis (BNST), the medial preoptic area (MPA) as well as the ventromedial hypothalamus (VMH) [10–12]. Lower expression levels have been reported for the hippocampus, the thalamus, the caudate putamen (CPu), the nucleus accumbens (Acb), the cingulate cortex, the cerebellum and the brain stem [10–12]. Overall, *Cyp19a1* mRNA expression has been shown to be higher in the brain of male rats, yet sex differences seem to vary by region [10–12]. However, these studies have been sparse and largely semi-quantitative, while analyzing gross anatomical regions [10–12]. Additionally, little is known about the identity of the neurons expressing aromatase [2]. In the avian system on the other hand, aromatase and *Cyp19a1* mRNA expression as well as the role of sex hormones have been extensively studied and considerable sex differences have been detected [13–16].

The present study sought to expand on existing *Cyp19a1* mRNA expression maps in mammals through systematic and quantitative cell imaging on histological sections of the male and female rat brain. By use of fluorescent in situ hybridization (FISH) combined with automated cell counting, the purpose of this study was to precisely define the distribution and quantify *Cyp19a1*-expressing cells throughout the limbic brain for the two sexes. Furthermore, the aim was to characterize the cells expressing *Cyp19a1* mRNA using double FISH to identify co-localization with specific markers for subtypes of neurons and glia cells.

## Methods

### Rat brain samples

All experiments were performed in conformity with the European Union laws and policies for use of animals in neuroscience research (European Communities Council Directive for the Care and the Use of Laboratory Animals, Regulation EU 2019/1010), and were approved by the local animal research committee. Sprague Dawley rats were housed in groups under standardized conditions at 22 ± 1 °C and a 12 h light/dark cycle, with food and water provided ad libitum. Female rats were selected at random estrous cycle phase.

**Fig. 1:**
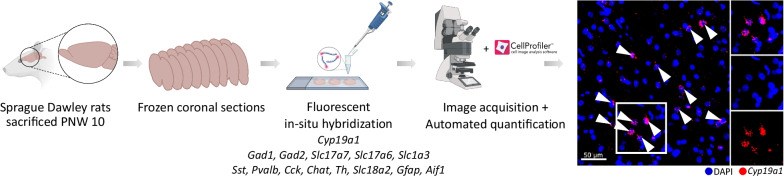
Design of the study. Brain sections of male and female rats killed at post-natal week 10 were analyzed by using gene-specific probes and performing fluorescent in situ hybridization. The slides were scanned with a digital full slide scanner at 20 × mean magnification. Quantification of *Cyp19a1*^+^ as well as cell marker^+^ cells was performed using CellProfiler. The right panel shows *Cyp19a1* (red) predominantly surrounding the DAPI-stained nucleus (blue) of the expressing cells. Some *Cyp19a1*^+^ nuclei are indicated with a white arrow. Abbreviations: PNW: post-natal week. The figure was created with BioRender.com

Brains from young adult (post-natal week (PNW) 10) male (*n* = 6) and female (*n* = 6) rats were quickly removed after decapitation and frozen at − 35°C in 2-methylbutane. Coronal frozen sections (16 µm) were prepared with a cryostat at − 20 °C, thaw-mounted onto poly-L-lysine-coated glass slides (Superfrost Plus, Menzel Glaser, Fisher Scientific, Braunschweig, Germany) and stored at − 80 °C until usage. The rat brains were cut in series of 20 slides (total slides = 180 per rat), each consisting of four to five sections from bregma 3.24 mm to -11.16 mm according to Paxinos and Watson [17]. A schematic overview of the study design is shown in Fig. 1.

### Single FISH

FISH was used to determine *Cyp19a1*-expressing cells, as described previously [18, 19]. For the single FISH experiments, every twentieth slide from the series was used for each rat, which corresponds to a section every 240 µm. Cryosections were air-dried, fixed in 4% paraformaldehyde and acetylated in 0.25% acetic anhydride/100 mM triethanolamine (pH 8). Sections were hybridized for 16 h at 65°C in 100 µl of formamide-buffer containing 1 µg/ml *Cyp19a1* digoxigenin-labeled riboprobe (DIG). The riboprobe for the *Cyp19a1* gene, covering all known mRNA transcript variants of the gene, was synthesized with DIG-labeled ribonucleoside triphosphate. Sections were washed at 65°C with saline–sodium citrate (SSC) buffers of decreasing strength and blocked with 20% goat serum and 1% blocking solution. DIG epitopes were detected with horseradish peroxidase (HRP) anti-DIG Fab fragments at 1:3000 and revealed using Cy3-tyramide at 1:100. Nuclear staining was performed with 4′ 6-diamidino-2-phenylindole (DAPI). Fluorophore-tyramides were synthetized as previously described [20].

### Double FISH

For the double FISH experiments, only the slides containing sections that revealed *Cyp19a1* mRNA expression in the single FISH experiments were used. The experimental procedure was the same as for the single FISH experiments, however, in addition to 1 µg/ml *Cyp19a1* DIG-labeled riboprobe, 1 µg/ml cell marker fluorescein-labeled riboprobe was added during the hybridization step. For this, synthesized with fluorescein-labeled ribonucleoside triphosphate, riboprobes with the complementary sequence of the transcripts for the following genes were used: glutamate decarboxylase 1 (*Gad1*), glutamate decarboxylase 2 (*Gad2*), glial high affinity glutamate transporter (*Slc1a3*), vesicular glutamate transporter 1 (*Slc17a7*), and vesicular glutamate transporter 2 (*Slc17a6*). Additionally, vesicular monoamine transporter (*Slc18a2*), tyrosine hydroxylase (*Th*), choline acetyltransferase (*Chat*), parvalbumin (*Pvalb*), somatostatin (*Sst*), cholecystokinin (*Cck*), glial fibrillary acidic protein (*Gfap*) and allograft inflammatory factor 1 (*Aif1*) were analyzed in one representative male and female rat. The reference sequences can be found in the supplementary material (Additional file 1: Table S1). Fluorescein epitopes were then detected with HRP conjugated anti-fluorescein antibody at 1:5000 and revealed using Cy2-tyramide at 1:250. HRP-activity was stopped by incubation of sections in 0.1 M glycine followed by a 3% H2O2 treatment. DIG epitopes were then detected as described in single FISH. The riboprobes that were used are shown in Additional file 1: Table S1.

### FISH image acquisition and analysis

All slides were scanned on a NanoZoomer 2.0-HT (RRID: SCR_021658) at 20 × mean magnification. Laser intensity and time of acquisition were set separately for each riboprobe. Images were analyzed and exported using the NDP.view software (version 2.6.17; Hamamatsu Photonics). Regions of interest (ROIs) were identified according to the rat brain atlas by Paxinos and Watson [17] and masked using Inkscape v0.92.4 (RRID:SCR_014479). The automated quantification of the number of *Cyp19a1*^+^ cells and amount of signal per nucleus was performed using CellProfiler Image Analysis Software (RRID:SCR_007358) [21]. The pipeline used in CellProfiler recognized and counted the DAPI-stained nuclei as well as the nuclei that were surrounded by Cy3- or Cy2-tyramide staining. Based on FISH experiments with the sense-probe of *Cyp19a1* a threshold for Cy3 and Cy2 was established (brightness < 0.3 and size < 3 pixels) to make sure that only staining was recognized that was clearly above background noise. For single FISH, the total number of nuclei per region was counted as well as the number of nuclei that were *Cyp19a1*^+^, and for each ROI the percentage of all *Cyp19a1*^+^ nuclei was calculated. As a first step, the entire ROI was evaluated and if sex differences were detected, the same analysis was performed for each of its sub-regions to determine which part of the region was driving the sex difference. If sub-regions showed a sex difference further division and analysis was performed. The size of the sub-regions differed, ranging from 0.12 mm (BNST sub-region) to 3 mm (cortical amygdala and hippocampus) coronally which accounted for 1–12 slices per region, while the number of nuclei ranged from 185 to 275,068. The hemispheres were analyzed separately but as no hemispherical differences were detected, the statistical analysis was performed on the combined values.

Regarding the double FISH experiments, only limbic regions with more than 0.8% *Cyp19a1*^+^ cells were included. If a region could be divided into sub-regions, only those with the most *Cyp19a1*^+^ cells were chosen and merged into one large sub-region to simplify the analysis. Co-localization was determined by the presence of the signals for both probes surrounding the same nucleus. All cells that were *Cyp19a1*^+^ were counted, as well as those that were co-expressing one of the cell markers and those that were positive for both *Cyp19a1* and the cell marker in question. The relative numbers were calculated referring to the number of cells that were positive for the cell marker and *Cyp19a1* in relation to all *Cyp19a1*^+^ cells in the region. As an exploratory study, only one representative animal from each sex was chosen to perform automated counting for the additional markers, due to the extent of the analyses. The co-expression pattern could not be investigated as the case of the CA2/3, in which, due to its morphology, the nuclei were very densely distributed, many times overlapping, and with highly expressed markers thus impeding the attribution of the signal to a single nucleus.

### Statistical analysis

For the single FISH experiments, the relative numbers of *Cyp19a1*^+^ nuclei per ROI were used for statistical analysis with JASP software (version 0.14.1, JASP Team, https://jasp-stats.org/) to compare the two groups (males vs females). For the double FISH, relative numbers of *Cyp19a1*^+^/cell marker^+^ nuclei per *Cyp19a1*^+^ nuclei in a ROI were used. Shapiro–Wilk tests were applied to assess normality of the data. As not all data sets showed normal distribution and considering the sample size of six males and six females, non-parametric testing was performed, to ensure unbiased results from outliers and the small sample size [22]. For each group, region and cell marker, the median in percent, the interval between the 25^th^ to 75^th^ percentile in percent, as well as the sample size were reported. The Mann–Whitney *U* test was used to compare the relative numbers of *Cyp19a1*^+^ nuclei per ROI between the two independent groups. As previous findings had shown that males had higher expression of *Cyp19a1* or that no significant sex differences could be detected, the expression of *Cyp19a1* was expected to be higher in males than in females, thus a one-tailed test was chosen [10–12]. The alternative hypothesis was that males had a larger relative number of *Cyp19a1*^+^ nuclei per ROI than females. A two-tailed Mann–Whitney *U* test was used to compare the sexes for the double FISH experiments. Statistical significance was defined as *p* ≤ 0.05 and the effect size was given by the rank-biserial correlation (r_rb_). Correction for multiple testing was performed by false discovery rate (FDR). Where no sex differences were found in the double FISH experiments, the male and female rats were merged into one group for illustration purposes.

## Results

### *Cyp19a1* mRNA expression in the limbic brain of young adult rats

**Fig. 2:**
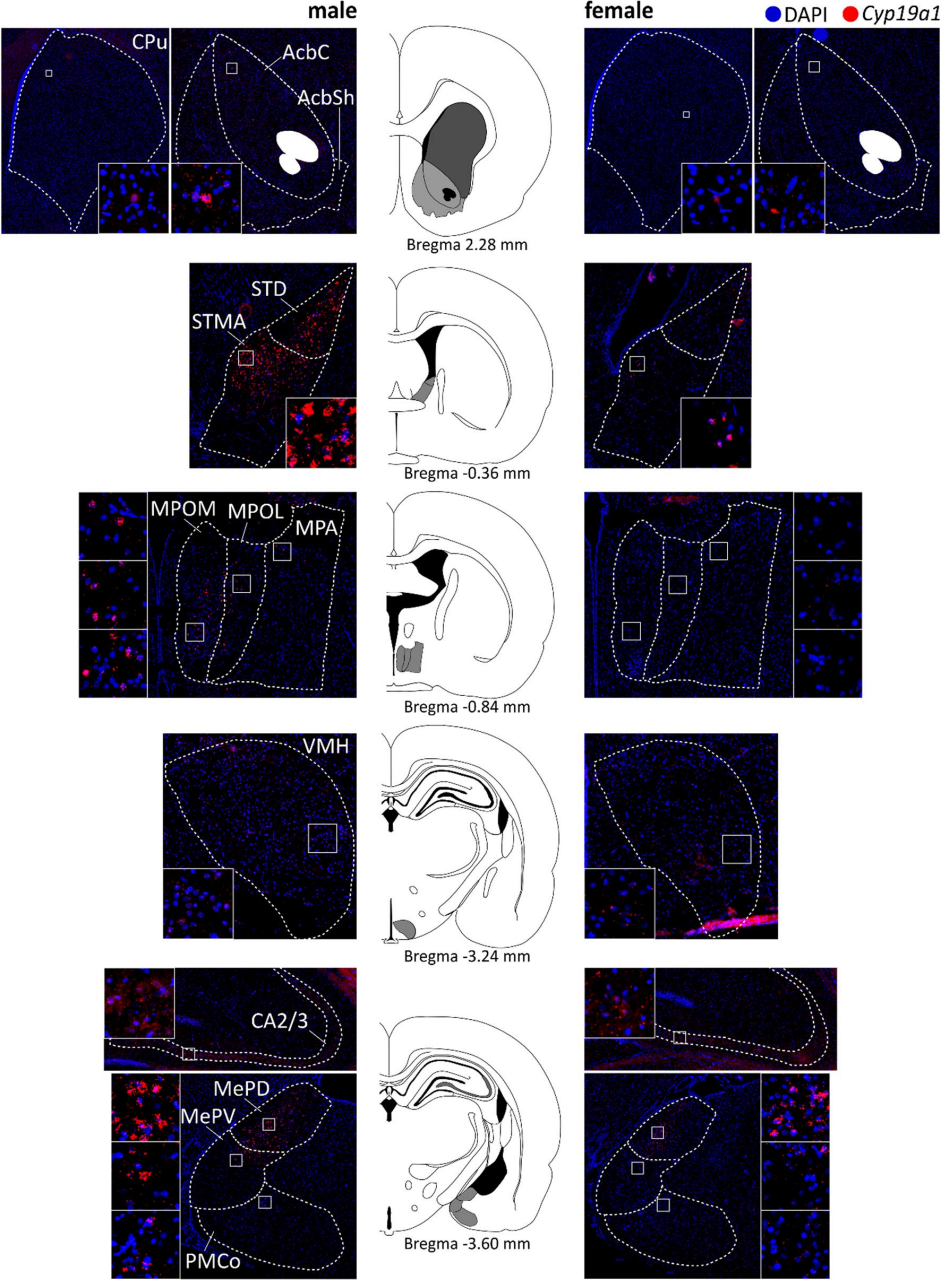
*Cyp19a1* mRNA expressing regions in the young adult male and female rat brain. Atlas figures based on Paxinos rat brain atlas 6th edition. *AcbC* nucleus accumbens core, *AcbSh* nucleus accumbens shell, *BNST* bed nucleus of the stria terminals, *CPu* caudate putamen, *MePD* posterodorsal part of medial amygdaloid nucleus: *MePV* posteroventral part of medial amygdaloid nucleus, *MPA* medial preoptic area, *MPOL* lateral part of medial preoptic nucleus, *MPOM* medial part of medial preoptic nucleus, *PMCo* posteromedial cortical amygdaloid nucleus, *STD* dorsal bed nucleus of the stria terminals, *STM* medial division of the bed nucleus of the stria terminals, *VMH* ventromedial hypothalamus

*Cyp19a1* mRNA was localized predominantly in the soma in proximity to the nucleus (Fig. 1). Mapping of *Cyp19a1* mRNA expression by single FISH revealed brain region-specific patterns, as shown in Fig. 2 and Table 1. Expression was observed in all investigated regions, namely the CPu, the Acb, the BNST, the hypothalamic regions, the cornu ammonis 2/3 fields of the hippocampus (CA2/3) and the amygdala.

**Table 1:** Sex differences in the percentage of *Cyp19a1*^+^ cells in different regions

Region			Sex	Descriptives			Mann–Whitney *U* test	
				Median	25–75th percentile	*N*	*p*	*r*_rb_
Nucleus accumbens			M	0.8	0.5–0.8	5	0.421	0.12
			F	0.5	0.5–0.6	5		
Caudate putamen			M	0.5	0.2–0.5	5	0.889	− 0.44
			F	0.5	0.5–0.7	5		
BNST	All		M	1.8	1.2–2.9	6	**0.047**	0.61
			F	1	0.6–1.3	6		
	STMA		M	3.1	2.3–5.0	6	**0.032**	0.67
			F	1.2	1.0–1.9	6		
	STMP		M	2.4	0.7–2.5	5	**0.026**	0.73
			F	0.3	0.2–0.5	6		
	STMV		M	0.8	0.7–0.9	6	0.294	0.22
			F	0.7	0.4–1.0	6		
	STD		M	0.8	0.4–14.5	6	0.409	0.11
			F	1.2	0.4–2.8	6		
	STLP		M	1	0.8–1.2	6	0.242	0.28
			F	0.7	0.4–1.2	6		
	STLI		M	0.7	0.3–1.1	6	0.287	0.22
			F	0.4	0.1–1.8	6		
	STLD		M	0.7	0.5–0.9	6	0.294	0.22
			F	0.6	0.1–1.0	6		
	STLV		M	0.4	0.2–0.6	6	0.650	− 0.11
			F	0.6	0.2–1.1	6		
Hypothalamic regions	All		M	1.4	0.9–2.5	6	0.047*	0.61
			F	0.8	0.4–1.1	6		
	MnPO		M	0.6	0.3–.5	6	0.350	0.17
			F	0.6	0.2–1.2	6		
	MPA		M	1.6	1.0–2.3	6	**0.047**	0.61
			F	0.7	0.3–1.3	6		
	MPOL		M	1.2	0.7–2.7	6	0.197	0.33
			F	0.8	0.3–1.4	6		
	MPOM		M	1.3	0.9–5.7	6	0.197	0.33
			F	1.2	0.4–2.1	6		
	Pe		M	0.4	0.3–1.0	6	0.374	0.14
			F	0.3	0.2–1.2	6		
	VMH		M	0.7	0.6–1.3	6	**0.021**	0.72
			F	0.4	0.4–0.5	6		
	VLPO		M	0.4	0.4–1.6	5	0.324	0.20
			F	0.6	0.1–1.0	6		
	VMPO		M	0.4	0.3–2.2	6	0.409	0.11
			F	0.9	0.2–1.6	6		
CA2/3 fields of hippocampus			M	2	1.2–2.7	6	0.008*	0.83
			F	0.6	0.5–0.7	6		
Amygdala	All		M	1.5	1.0–1.6	6	0.004*	0.89
			F	0.6	0.5–0.7	6		
	Medial nucleus	All	M	3.8	2.2 -5.4	6	0.004*	0.89
			F	1.1	0.7–1.5	6		
		MeAD	M	0.5	0.5–0.6	6	0.021*	0.72
			F	0.2	0.1–0.4	6		
		MeAV	M	0.7	0.6–0.8	6	0.005*	1
			F	0.2	0.2–0.3	4		
		MePV	M	1.4	0.9–2.4	6	0.013*	0.78
			F	0.6	0.4–0.7	6		
		MePD	M	7.1	3.4–10.0	6	0.008*	0.83
			F	1.6	1.0–2.4	6		
	Cortical nucleus		M	1	0.6–1.6	6	0.242	0.28
			F	0.8	0.5–1.1	6		
	Basal nucleus		M	0.6	0.4–0.7	6	0.12	0.44
			F	0.4	0.2–0.5	6		
	Central nucleus		M	0.3	0.3–0.5	6	0.531	0
			F	0.4	0.2–0.6	6		
	Lateral nucleus		M	0.6	0.4–0.8	6	0.090	0.50
			F	0.4	0.2–0.5	6		
	Intraamygdaloid division of BNST		M	0.9	0.8–0.9	6	**0.047**	0.61
			F	0.3	0.1–0.6	6		
	Amygdalo-hippocampal area	All	M	1.7	1.2–2.1	6	0.013*	0.78
			F	0.6	0.4–0.9	6		
		AHiAL	M	2.1	1.5–2.2	6	0.002*	0.94
			F	0.5	0.5–0.7	6		
		AHiPL	M	0.3	0.2–0.9	4	0.548	0
			F	0.4	0.3–0.5	5		
		AHiPM	M	2	1.4–2.7	4	0.143	0.50
			F	0.5	0.5–1.6	5		

The sex differences in the main regions of interest and their sub-regions are presented as the percentage of number of *Cyp19a1*^+^ nuclei throughout the male and female rat brain. *p*: uncorrected *p*-value of the Mann–Whitney U test, bold indicates an uncorrected* p* < 0.05, an asterisk indicates a significant p-value after correction for multiple comparison by false discovery rate. *r*_rb_: effect size based on rank-biserial correlation. AHiAL: anterolateral part; AHiPL: posterolateral part; AHiPM: posteromedial part; MeAD: anterodorsal part; MeAV: anteroventral part; MePD: posterodorsal part; MePV: posteroventral part; MnPO: median preoptic nucleus; MPA: medial preoptic area; MPOL: medial preoptic nucleus, lateral part; MPOM: medial preoptic nucleus, medial part; Pe: periventricular hypothalamic nucleus; STD: dorsal part; STLD: lateral division, dorsal part; STLI: lateral division, intermediate part; STLJ: lateral division, juxtacapsular part; STLP: lateral division, posterior part; STLV: lateral division, ventral part; STMA: medial division, anterior part; STMP: medial division, posterior part; STMV: medial division, ventral part; VLPO: ventrolateral preoptic nucleus; VMH: ventromedial hypothalamic nucleus; VMPO: ventromedial preoptic nucleus

**Fig. 3:**
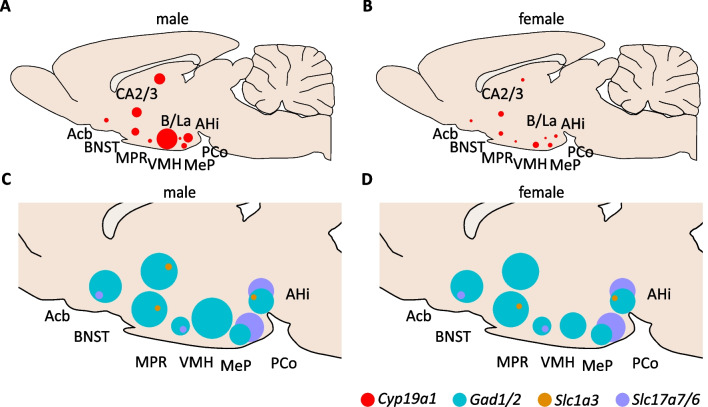
Schematic distribution of *Cyp19a1*^+^ cells and co-expression with cell markers. Overview of *Cyp19a1*^+^ cells in the male (**A)** and female (**B)** young adult limbic rat brain. The size of the circles is relative to the percentage of *Cyp19a1*^+^ cells in the ROI. In males the overall percentage was higher than in females with significant sex differences in the BNST, MPR, VMH, CA2/3, MeP and AHi. The lower panel gives an overview of the percentage of cells co-expressing *Gad1* and *Gad2* (turquoise), *Slc17a7* and *Slc17a6* (purple) and *Slc1a1* (orange) together with *Cyp19a1* in relation to all *Cyp19a1*^+^ cells in the ROI in males (**C)** and females (**D)**. In most regions the majority of *Cyp19a1*^+^ cells were GABAergic (*Gad1/2*^+^) except for the AHi and PCo where the majority were glutamatergic (*Slc17a7/6*^+^). The size of some circles is exaggerated for visibility and therefore is not always proportional to the actual percentage (see Additional file 1: Table S2). *Acb* nucleus accumbens, *AHi* amygdalo-hippocampal nucleus, *B/LA* basal and lateral amygdaloid nuclei, *BNST* bed nucleus of the stria terminalis, *MeP* posterior medial amygdaloid nucleus, *MPR* medial preoptic region, *PCo* posterior cortical amygdaloid nucleus, *VMH* ventromedial hypothalamic nucleus

A schematic overview of *Cyp19a1* expression in the male and female rat brain (Fig. 3A, B) points out the prevalence of *Cyp19a1*^+^ cells in the brain structures of interest. The highest percentage of cells expressing *Cyp19a1* was found in the BNST, followed by the amygdala, the hypothalamic regions as well as the CA2/3. Lower expression was noted in the Acb and the CPu.

### Sex differences in the expression of *Cyp19a1* mRNA

**Fig. 4:**
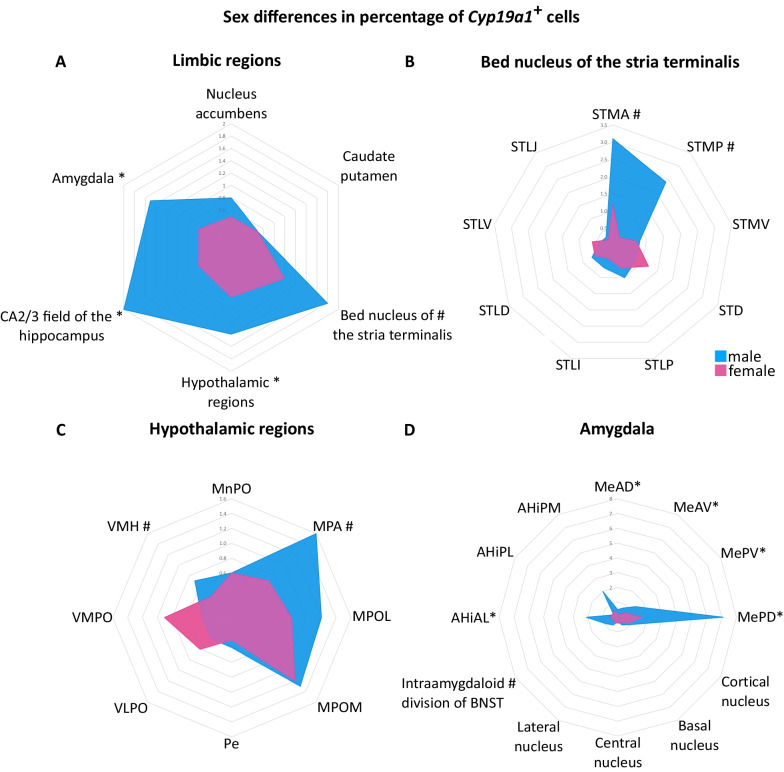
Sex differences in the percentage of *Cyp19a1*^+^ nuclei in the ROIs and their sub-regions. Represented are the medians of the percentages of *Cyp19a1*^+^ cells. ^#^p < 0.05 before FDR; *p < 0.05 after FDR. *AHiAL* anterolateral part of amygdalo-hippocampal nucleus, *AHiPL* posterolateral part of amygdalo-hippocampal nucleus; *AHiPM* posteromedial part of amygdalo-hippocampal nucleus, *MeAD* anterodorsal part, medial amygdaloid nucleus; *MeAV* anteroventral part, medial amygdaloid nucleus, *MePD* posterodorsal part, medial amygdaloid nucleus, *MePV* posteroventral part, medial amygdaloid nucleus, *MnPO* median preoptic nucleus, *MPA* medial preoptic area; MPOL: medial preoptic nucleus, lateral part, *MPOM* medial preoptic nucleus, medial part, *Pe* periventricular hypothalamic nucleus, *STD* dorsal part of BNST, *STLD* lateral division, dorsal part of BNST, *STLI* lateral division, intermediate part of BNST, *STLJ* lateral division, juxtacapsular part of BNST; *STLP* lateral division, posterior part of BNST, *STLV* lateral division, ventral part of BNST, *STMA* medial division, anterior part of BNST, *STMP* medial division, posterior part of BNST, *STMV* medial division, ventral part of BNST, *VLPO* ventrolateral preoptic nucleus, *VMH* ventromedial hypothalamic nucleus; VMPO: ventromedial preoptic nucleus

Significant sex differences were detected in the BNST, the hypothalamic regions, the CA2/3 as well as in the amygdala with effect sizes ranging from 0.61 to 0.89. In these areas, the males had two to three times higher percentage of *Cyp19a1*^+^ cells relative to females. After FDR correction, significant sex differences remained in the amygdala and the CA2/3. No sex differences were found in the Acb and the CPu (Fig. 4, Table 1).

#### Bed nucleus of the stria terminalis

The sub-regions with the highest percentage of *Cyp19a1*^+^ cells in the BNST were the anterior and posterior medial divisions (STMA and STMP) with a median of up to 3% of the cells being positive for the marker. These were also the only sub-regions in which a sex difference was detected (r_rb_ = 0.67 and 0.73, respectively), with males showing a higher percentage. In the remaining sub-regions of the BNST with a lower percentage of *Cyp19a1*^+^ cells (< 1%; Table 1) no sex differences were noted.

#### Hypothalamic regions

Within the hypothalamus, the highest percentage of *Cyp19a1*^+^ cells was observed in the MPA as well as the lateral and medial parts of the medial preoptic nucleus (MPOL and MPOM) with up to 1.6% of cells expressing *Cyp19a1*. While no sex differences were detected in the MPOL and MPOM, males had more than two times more *Cyp19a1*^+^ cells in the MPA than females (*r*_rb_ = 0.61).

A similar sex difference was observed in the VMH (*r*_rb_ = 0.72), although the percentage of *Cyp19a1*^+^ cells in this region was lower (see Fig. 4, Table 1).

#### Amygdala

The sub-region with both the highest percentage of *Cyp19a1*^+^ cells as well as the most pronounced sex differences in the amygdala was the medial amygdaloid nucleus (Table 1). Here, males had 3.5 times more *Cyp19a1*^+^ cells than females (*r*_rb_ = 0.89) and in the posterodorsal part (MePD) almost 4.5 times more (*r*_rb_ = 0.83) with 7% *Cyp19a1*^+^ cells.

Additionally, sex differences were detected within the amygdalo-hippocampal area (AHi; *r*_rb_ = 0.78; Table 1) as well as the intra-amygdaloid division of the BNST (STIA; *r*_rb_ = 0.61). For the AHi, the sex differences were driven by the anterolateral part (AHiAL; *r*_rb_ = 0.94) in which the percentage of *Cyp19a1*^+^ cells was four times higher in males. On the other hand, within the other sub-regions of the amygdala no sex differences were found (Fig. 4, Table 1).

### Characterization of *Cyp19a1*-expressing cells

**Fig. 5:**
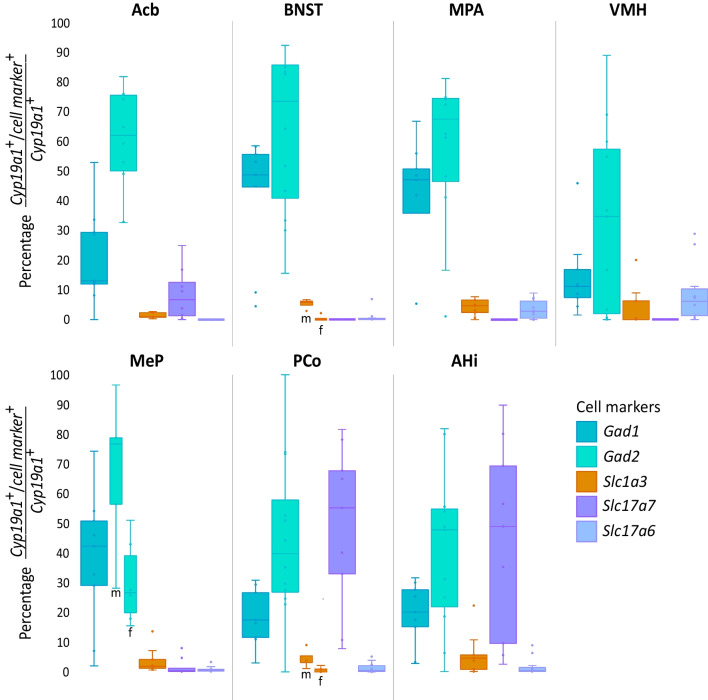
Co-expression of cell markers with *Cyp19a1* in different brain regions. Abbreviations: Acb: nucleus accumbens; AHi: amygdalo-hippocampal nucleus; BNST: bed nucleus of the stria terminalis; f: female; m: male; MeP: posterior part of the medial amygdaloid nucleus; MPR: medial preoptic region; PCo: posterior part of the cortical amygdaloid nucleus; VMH: ventromedial hypothalamic nucleus

**Fig. 6:**
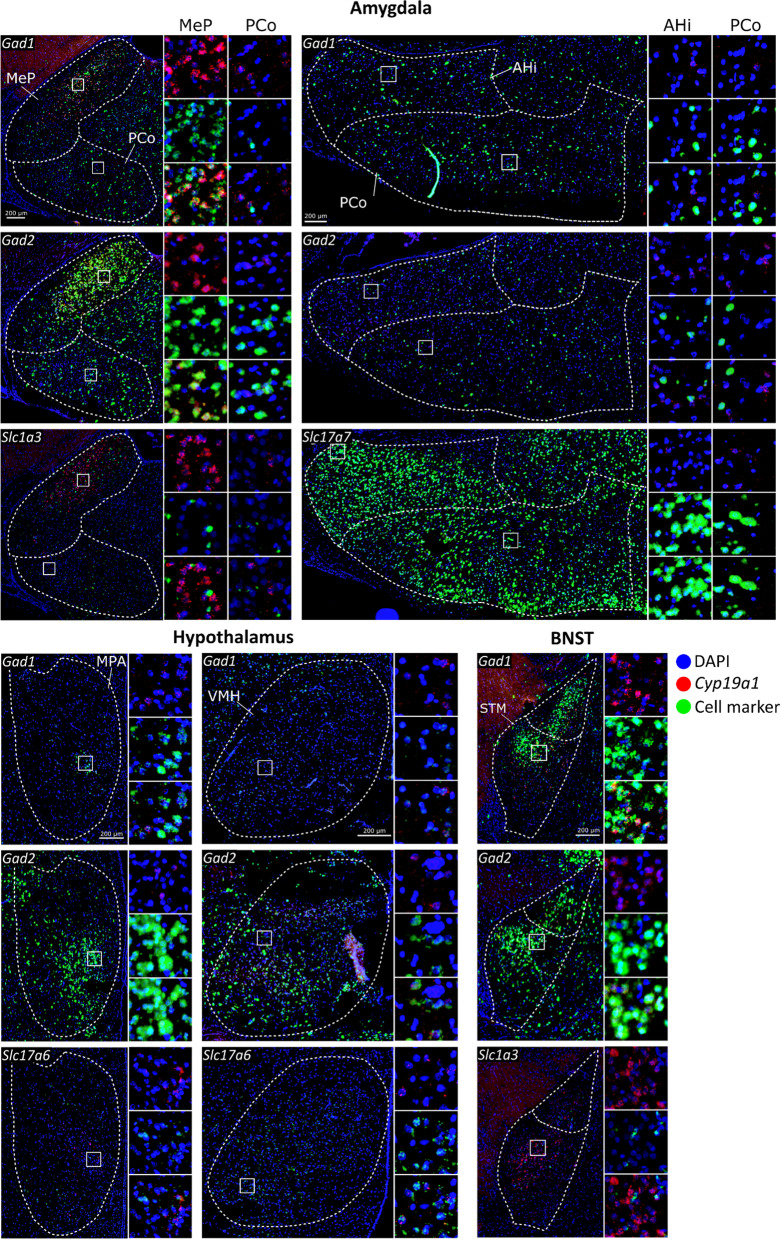
Representative co-expression profile of *Cyp19a1* and cell markers. As expression of *Cyp19a1* was higher in males, images from one male rat were chosen as representatives. In the left upper corner of the images that show the region of interest, the cell marker (green) that the double FISH experiment was performed with is indicated. Only images of cell markers that showed co-expression with *Cyp19a1* that can be detected by eye in that specific region are shown. The magnified images on the right of the images with the whole region show DAPI + *Cyp19a1* (top), DAPI + cell marker (middle), DAPI + *Cyp19a1* + cell marker (bottom). *AHi* amygdalo-hippocampal nucleus, *BNST* bed nucleus of the stria terminals, *MeP* posterior medial amygdaloid nucleus, *MPA* medial preoptic area, *PCo* posterior cortical amygdaloid nucleus, *STM* medial division of the bed nucleus of the stria terminals, *VMH* ventromedial hypothalamus

An overview of the results of the co-expression double FISH experiments is shown in Figs. 3C, D and 5, the co-FISH images in Fig. 6, while Table 2 shows all results for each marker. Despite the variation of the ranges between males and females (Table 2), very few sex differences were observed, and none retained significance after FDR correction.

**Table 2:** Co-expression of cell markers with *Cyp19a1* in different brain regions

Region		Acb		BNST		MPR		VMH		MeP		PCo		AHi	
Sex		M	F	M	F	M	F	M	F	M	F	M	F	M	F
*Gad1* co-localization															
Median		28.6	10.3	53.1	27	47	46.3	11.8	5.9	42.2	26.4	17.4	18.8	20	21.4
25th percentile		13	6.1	48.7	7.9	35.8	32.7	11.1	3.6	32.7	5.7	16.4	8.9	15.1	13.8
75th percentile		33.6	16.7	55.6	48.2	48.6	54.7	16.8	11	54.1	47.1	17.5	27.7	30	25.9
*N*		5	4	5	4	5	4	5	4	5	4	5	4	5	4
Mann–Whitney *U* test	*p*	0.19		0.286		0.905		0.19		0.413		0.905		0.73	
	*r*_b_	0.6		0.5		− 0.1		0.6		0.4		0.1		0.2	
*Gad2* co-localization															
Median		53	74.1	84.2	38.3	73.4	44.7	59.9	8.6	76.6	26.6	51.8	28.6	54.8	25
25th percentile		49	59.2	82.8	30.8	65	22.8	34.6	0.2	56.4	19.8	39.1	23.9	50.1	18.6
75th percentile		64.8	76	87.2	59	74.7	67.7	69	31.7	78.8	39	68.7	40.6	73.9	31.1
*N*		5	5	6	6	6	6	5	6	5	6	6	6	6	5
Mann–Whitney *U* test	*p*	0.421		0.065		0.132		0.082		**0.017**		0.132		0.052	
	*r*_b_	− 0.4		0.7		0.6		0.7		0.9		0.6		0.7	
*Slc17a7* co-localization															
Median		10.3	1							0.3	2.3	40.1	73	35.3	74.7
25th percentile		8.1	0.3							0.06	0	33	53.5	9.5	52.6
75th percentile		14.6	5.4							0.4	5.4	55.2	79	48.9	82.5
*N*		4	4							5	4	5	4	5	4
Mann–Whitney *U* test	*p*	0.2								0.901		0.19		0.286	
	*r*_b_	0.6								− 0.1		− 0.6		− 0.5	
*Slc17a6* co-localization															
Median				0.3	0	3	1.8	4.9	7.2	0.8	0	1	0.4	0.7	0.1
25th percentile				0	0	2.7	0	1.5	0.5	0	0	0.5	0	0	0
75th percentile				0.4	0	4.1	7.3	7.8	11.1	0.9	0	2.4	0.5	1.4	1.1
*N*				5	5	5	5	5	5	5	5	5	5	5	5
Mann–Whitney *U* test	*p*			0.48		1		1		0.48		0.398		1	
	*r*_b_			0.3		0		0		0.3		0.4		0	
*Slc1a3* co-localization															
Median		1.6	0.5	5.7	0	5.8	2.4	7.6	0	4.5	1.1	3.9	0.3	5.2	0.8
25th percentile		0.9	0.4	4.8	0	5	0.6	4.7	0	2	0.6	3.1	0	4.5	0
75th percentile		2.4	1.4	6.1	0	6.9	3.1	11.7	0	8.7	1.6	5.3	0.9	6.9	4.5
*N*		4	3	4	5	4	5	4	5	4	5	4	5	4	5
Mann–Whitney *U* test	*p*	0.229		**0.015**		0.111		0.081		0.063		**0.037**		0.268	
	*r*_b_	0.7		1		0.7		0.7		0.8		0.9		0.5	

The sex differences in the main regions of interest are presented as the percentage of *Cyp19a1*^+^/cell marker^+^ nuclei from all *Cyp19a1*^+^ nuclei in a region. As no *Slc17a7* was expressed in the BNST, MPR or VMH nor *Slc17a6* in the Acb, no co-expression analyses were performed in those regions. *p*: uncorrected *p*-value of the Mann–Whitney *U* test, bold if uncorrected *p* < 0.05. *r*_rb_: effect size based on rank-biserial correlation. *Acb* nucleus accumbens; *AHi* amygdalo-hippocampal nucleus, *BNST* bed nucleus of the stria terminalis, *f* female, *m* male; *MeP* posterior part of the medial amygdaloid nucleus, *MPR* medial preoptic region, *PCo* posterior part of the cortical amygdaloid nucleus, *VMH* ventromedial hypothalamic nucleus

In all analyzed regions, expression of *Cyp19a1* mRNA was predominant in GABAergic cells, indicated by co-expression with either *Gad1* or *Gad2*. In almost all regions, co-expression with *Gad2* was higher than with *Gad1*, especially within the male rat brain. Cells co-expressing a glutamatergic marker (*Slc17a7* or *Slc17a6*) were also detected in some regions, however, with few exceptions, in much lower percentages. Additionally, double FISH with *Slc1a3* indicated *Cyp19a1* expression in astrocytes in some regions.

### GABAergic markers

#### Gad1

Within the Acb and VMH, less than 15% of *Cyp19a1*^+^ cells were *Gad1*^+^, while it was almost 50% within the BNST and the medial preoptic region (MPR = MPA + MPOM + MPOL). The sub-region with the highest co-expression within the amygdala was the posterior medial amygdaloid nucleus (MeP) with about 40% of *Cyp19a1*^+^ cells. In the posterior cortical amygdaloid nucleus (PCo) and AHi expression of *Gad1* was detected in about 20% of *Cyp19a1*^+^ cells. With effect sizes between 0.5 and 0.6, a trend towards higher *Gad1* co-expression with *Cyp19a1* was observed in the Acb, BNST and VMH of males compared to females.

#### Gad2

Overall, *Gad2* was the cell marker that showed the highest co-expression with *Cyp19a1* throughout most of the investigated brain regions. Within the Acb, about 60% of the *Cyp19a1*^+^ cells were expressing *Gad2,* higher levels were found in the BNST and MPR with about 70% co-expression, whereas lower levels were noted in the VMH (35%). Although no sex differences were statistically significant, the effect sizes (r_rb_ = 0.6–0.7) were pointing towards more *Gad2*^+^/*Cyp19a1*^+^ cells in the male BNST and hypothalamic regions than in females.

The only significant sex difference was found for *Gad2* within the MeP, were the co-expression was almost 80% in males and about 30% in the females with an effect size of 0.87. In the other amygdaloid nuclei (PCo and AHi) the effect size (*r*_rb_ = 0.7) were indicating the same trend, and the co-expression levels were between around 35 and 50%.

### Glutamatergic markers

#### Slc17a7

No co-expression of *Slc17a7* and *Cyp19a1* was detected within the BNST and the hypothalamic regions. Within the Acb, some co-expression with *Cyp19a1* was found (7%), while in the amygdala, the co-expression was predominant in the PCo and AHi with 50–55%. Although not statistically significant, effect sizes between − 0.5 and − 0.6 were indicating a higher co-expression of *Slc17a7* and *Cyp19a1* in these regions in females than in males. In the MeP on the other hand, very low (3%) co-expression was detected.

#### Slc17a6

For this cell marker, the Acb did not show co-expression with *Cyp19a1.* Within the BNST and amygdala, no or very low co-expression was observed with *Cyp19a1,* while low levels were detected in the MPR (3%) and VMH (6%).

### Astrocytic marker

#### Slc1a3

Low co-expression with this marker was detected and in most regions no sex differences were observed. Very low co-expression of 1% was detected within the Acb and while the MPR showed co-expression of 5%, none could be observed within the VMH. Notably, no co-expression was found in the BNST of the female rats while about 6% could be detected in the males (*r*_b_ = 0.9). Within the amygdala, no co-expression was identified in the MeP while slightly higher levels were found in the AHi (5%).

### Exploratory investigation of additional markers

When testing for co-expression with markers for interneurons, parvalbumin mRNA (*Pvalb*) did not seem to be co-expressed with *Cyp19a1*, while some co-expression could be seen with the transcripts of somatostatin (*Sst*) and cholecystokinin (*Cck*). For both the male and the female, *Cck* was co-expressed the most in cells of the amygdala with sub-regional sex differences, which could also be seen for *Sst*, while there were indications pointing to a higher co-expression of *Sst* in females in the BNST and VMH.

None of the monoaminergic markers (*Slc18a2* and* Th*) or the cholinergic marker (*Chat*) were co-expressed in a significant way with *Cyp19a1* in the regions examined. The astrocytic marker *Gfap* seemed to be co-expressed at similar levels as the other astrocytic marker *Slc1a3*; the microglial marker *Aif1* seemed to be co-expressed at low levels of 1–3% in the BNST, VMH and PCo in the male, while there was an indication of a higher co-expression with this marker in the female, especially in the MeP. More information on the additional markers can be found in Additional file 1: Table S2.

## Discussion

The present findings provide a quantitative map of the distribution and identity of *Cyp19a1*^+^ cells in the limbic brain of male and female rats*.* Overall, the percentage of *Cyp19a1*^+^ cells was higher in the male rat brain with sex differences detected in several regions, namely in the amygdala, the BNST, the CA2/3 and the hypothalamic regions. GABAergic markers were expressed in the majority of the *Cyp19a1*^+^ cells in most regions, with the exception of the PCo and AHi where the highest co-expression was found in glutamatergic cells. A smaller fraction of cells co-expressed *Slc1a3,* especially in the amygdala suggesting expression of *Cyp19a1* also in astrocytes. Moreover, region- and gene-specific sex differences were detected regarding co-expression.

### Distribution and sex differences

The present findings confirm and extend previous results obtained in the rat brain by qPCR [10], colorimetric ISH [11, 12] and RNase protection assay [12, 23]. Moreover, the localization pattern of *Cyp19a1*^+^ cells detected in this study discretely overlaps with the aromatase protein levels in areas of high expression, suggesting that brain *Cyp19a1* is regulated transcriptionally [24]. The chosen regions of interest emerged as relevant in the present study, based on the results in the above-mentioned studies. However, while these studies focused on the overall expression throughout gross anatomical regions, an in-depth quantitative analysis of the sub-regions was here performed as sub-regions vary both morphologically as well as functionally [25].

The current findings show that overall *Cyp19a1*^+^ cells were more numerous in the brain of male rats, yet sex differences were found to vary by region. This confirms previous findings that have shown either significantly higher or a trend towards higher expression in males [10–12]. Similar sex differences have been reported in birds [13, 26]; and, interestingly, Naftolin et al. pointed to the comparability of the avian and rodent limbic system and hypothalamic nuclei even though other parts of the avian brain may diverge [14, 15]. It should also be noted that, in the present study, the variance in the percentage of *Cyp19a1*^+^ cells among the males was higher than the females. This could explain why sex differences have not been detected in studies of rats based on small samples [10, 12] or unknown number of animals [11]. Upon binding of testosterone to its receptor, androgens have been reported to be able to act as transcription factors to influence the transcription of *Cyp19a1* [23]. Therefore, the high levels and large variance in aromatase expression for males might indeed be driven by inter-individual differences in testosterone levels. Since the current study did not reveal a large variance in the percentage of *Cyp19a1*^+^ cells between females that have been killed at random estrus cycle days, it can be assumed that the cyclic fluctuation of estrogens only has a small to no effect on the expression of *Cyp19a1* in the brain, as shown in humans [27]. However, one study points to aromatase activity, but not protein expression, varying depending on estrous cycle phase in rodents [28]. This calls for further investigation on the expression of *Cyp19a1* while considering estrous cycle phase. Testosterone levels on the other hand largely vary between males, depending on their hierarchical status in the group [29, 30]. There is also evidence that the effect of gonadal hormones on *Cyp19a1* transcription is more pronounced in males than in females [31]. Interestingly, in the present study, sex differences in the percentage of *Cyp19a1*^+^ cells have been found in many sexually dimorphic regions of the rat brain that contain a high number of gonadal hormone receptors [25].

To date, there has not been a consensus on which region presents the highest percentage of *Cyp19a1*^+^ cells in the rat brain. Here, when parcellated into sub-regions based on the rat brain atlas by Paxinos and Watson [17], the MePD of the male rats showed the highest percentage of *Cyp19a1*^+^ cells, followed by the STMA and STMP to which most of the BNSTs *Cyp19a1*^+^ cells seem to be confined. In the female brain, the MePD and STMA were also the sub-regions with the highest percentage of *Cyp19a1*^+^ cells, while very few were found in the STMP. Unlike previous findings that were inconsistent about whether there is a sex difference in *Cyp19a1* expression in these regions or not, a distinct sex difference was observed in the present study. This indicated that males had a higher percentage of *Cyp19a1*^+^ cells both in the BNST, specifically the STMA and STMP, as well as within the MeP, especially in the MePD where the males had levels up to 4 times higher than females. Both the BNST and MeP are distinct sexually dimorphic regions of the rat brain, being larger in size in males than in females [25, 32]. They are strongly interconnected with different hypothalamic nuclei such as the MPR and the VMH, and contain a large density of gonadal hormone receptors [25]. Additionally, these sub-regions of the BNST and MeP have been associated with sex-specific behaviors such as parental care, aggression, and sexual behavior, as well as other social behaviors that might be associated with sex differences in *Cyp19a1* expression [25, 32].

A region that, to our knowledge, has never been analyzed in connection to *Cyp19a1* expression in rats was the AHi. Especially within the male AHiAL, the AHi displayed a very high percentage of *Cyp19a1*^+^ cells, comparable to those of the BNST, while the percentage in the female AHi was three times lower. This sub-region of the amygdala has been repeatedly associated with socio-sexual behaviors in rodents and is connected to various regions that express high levels of *Cyp19a1* such as the MeP, BNST and MPR [33]. Additionally, the AHi is enriched with gonadal hormone receptors, especially the estrogen receptor alpha [33]. Particularly in males, another region with a high percentage of *Cyp19a1*^+^ cells was the CA2/3. The hippocampus has been so far rated lower in *Cyp19a1* expression levels than the hypothalamic regions [10–12]. This could be due to the analysis of the hippocampus as a whole region that might have diluted the results. As Wagner and Morrell showed using in situ hybridization, the *Cyp19a1* expression in the hippocampus seems to be restricted to the CA2/3 [11], and therefore was the only sub-region of the hippocampus that was analyzed here. The role of aromatase in the hippocampus is still not clear, although local estrogen production in the hippocampus might play a role in memory and learning [8]. Short-term spatial memory tests in aromatase knockout mice, for example, indicated that both sexes display cognitive deficits [34]. Contrary to the findings of this study, where the percentage of *Cyp19a1*^+^ cells were three times higher in males than in females, previous studies have not found any sex differences within the hippocampus [10, 35].

In both sexes, the percentage of *Cyp19a1*^+^ cells in the MPR were similar and, in agreement with previous studies in rats, rather low compared to the BNST and MeP [10–12]. With the division into sub-regions, the current study could additionally confirm that males have a two times higher percentage of *Cyp19a1*^+^ cells within the MPA, however not within the MPOL and MPOM. Similar results have been found in homologous brain regions in the Japanese quail [13–15, 26]. The MPR is another one of the sexually dimorphic regions on several levels (volume, neuropeptide expression, neurotransmitters). It is highly sensitive to gonadal hormones, not only during development, but also throughout adulthood, and it is involved in behaviors that show consistent sex differences such as maternal and sexual behavior [36].

Interestingly, the current study displayed rather lower percentages of *Cyp19a1*^+^ cells for the VMH, which contradicts the results from previous studies where the VMH has been reported to have similar expression levels to the MPR [11, 12]. These divergences may be explained by the fact that the relative number of *Cyp19a1*^+^ nuclei was quantified in this study, and that the results would have been different if the total amount of *Cyp19a1* mRNA in each region would be quantified as done in other studies [11, 12]. The present findings of almost two times more *Cyp19a1*^+^ cells within the VMH of males than of females, however, match the ones by Wagner and Morrell [11]. The VMH shares connections with many of the other sexually dimorphic regions and is itself sexually dimorphic by having a higher synapse density and a larger volume in males compared to females [25]. Additionally, this region contains a high number of gonadal hormone receptors [25].

Furthermore, very low percentages of *Cyp19a1*^+^ cells in the CPu as well as the Acb were observed in the current study, which received little attention in previous research on aromatase [11] but might be of relevance to addiction, as a blocking effect of nicotine on aromatase has been demonstrated [37, 38]. Interestingly, rather sparsely and evenly distributed, *Cyp19a1*^+^ cells with high expression were seen throughout both regions.

### Characterization

The current results point to region-specific identity of cells expressing *Cyp19a1*. Consistent with studies in the quail, rodent, and human brain, *Cyp19a1* expression was found in GABAergic neurons [26, 39, 40]. Indeed, a large number of *Cyp19a1*^+^ cells in all investigated regions was noted to co-express *Gad1* and/or *Gad2*. Interestingly, *Gad2* was co-expressed more frequently than *Gad1*, which suggests that at least part of the GABAergic neurons expressing *Cyp19a1* were *Gad1*^*−*^ while co-expressing *Gad2*. *Gad1* and *Gad2* code for two isoforms of the enzyme glutamate decarboxylase (GAD67 and GAD65, respectively) that catalyzes the rate-limiting step in GABA synthesis [41]. GAD67 appears to be constitutively active and thereby providing a constant availability of GABA [42]. GAD65 on the other hand seems to be responsible for rapid production of large amounts of GABA in neurons in response to presynaptic activity [41, 42]. This might suggest that *Cyp19a1* is expressed in GABAergic neurons that are able to impact synaptic plasticity through short-term high firing patterns. Furthermore, aromatase is not only localized in the soma, but also in dendrites, axons and the presynaptic buttons of neurons [43]. These findings provide evidence that aromatase and brain-derived estradiol might play a role in the regulation of the expression of synaptic proteins as well as neurotransmitter levels in these neurons [7, 44, 45]. Furthermore, although only few statistically significant sex differences in the co-expression could be detected, the overall trend was that the males had more *Cyp19a1*^+^/*Gad*^+^ cells in all ROIs. A similar sex difference has been previously described in Japanese quails [26].

In addition to the detection of *Cyp19a1* in GABAergic neurons, co-expression with glutamatergic markers was observed as well, however not as frequently. Although glutamatergic neurons have been reported to express *Cyp19a1* in the neocortex and hippocampus of humans and monkeys [39, 46], to our knowledge this is the first time that regional identification of *Cyp19a1*^+^ glutamatergic neurons is provided in rats. Not surprisingly, in different regions, *Cyp19a1* was co-expressed with either *Slc17a7* or *Slc17a6,* as their expression is distributed in a complementary pattern throughout the adult rat brain [47]. Interestingly, the regions with high *Slc17a7* co-expression, AHi and PCo, are considered to be cortical-like structures of the amygdala [33]. *Cyp19a1* has previously been found to be expressed by glutamatergic neurons in cortical or cortical-like structures in the mammalian brain [43, 48]. It has been suggested that the expression of *Cyp19a1* in both glutamatergic as well as GABAergic neurons could mean that estrogens play a role in balancing excitation and inhibition in the brain [9]. Additionally, in contrast to GABAergic markers, co-expression with *Slc17a7* seemed to be higher in the female amygdala than in the male, although not reaching statistical significance.

Another cell type that has been previously connected to *Cyp19a1* expression in mammals is astrocytes [9, 39]. To our knowledge, expression of *Cyp19a1* in glia cells in adult rodents has only been shown upon brain injury, when *Cyp19a1* is expressed in reactive astrocytes [49, 50]. Indeed, it is possible that the increase of aromatase expression after brain injury might be solely due to its expression in cells of the astroglial lineage in the injured brain area [51]. A local increase of aromatase expression in astrocytes after brain injury has also been shown in zebra finch [52, 53]. However, in humans, *CYP19A1* expression has also been identified in a subpopulation of astrocytes in the temporal cortex in the healthy brain [39]. The current findings indicate the presence of a small population of astrocytes (*Slc1a3*^+^) that express *Cyp19a1* in several brain regions in rats, although predominantly in the males. The role of *Cyp19a1* expression in astrocytes under physiological conditions remains however unknown.

The present pilot analyses testing different GABA-interneuron markers indicate that *Cck*- and *Sst*- but not *Pvalb*- expressing interneurons are expressing *Cyp19a1*. *Cck*-interneurons in the amygdala and Sst-interneurons in limbic regions, where the current study found indication for co-expression with *Cyp19a1*, might play a role in the response and modulation of anxiety in rodents [54, 55]. Finally, the observed widespread trend regarding a higher percentage of co-expression with GABAergic and *Slc17a7*^+^ markers in males compared to females, except in the PCo and AHi where the opposite trend was observed, calls for further investigations.

### Perspectives and significance

Several of the detected sex differences in the percentage of *Cyp19a1*^+^ cells were observed in regions known to be sexually dimorphic, showing a high density of gonadal hormone receptors, and often linked to social, sex-specific, and affective behaviors. There is a possibility for translatability to humans as *Cyp19a1* and aromatase protein are expressed in similar regions (e.g., amygdala and preoptic area) in the human brain [27, 56, 57]. Based on the results of this study, further investigation is needed to understand the role of *Cyp19a1* expression in the limbic brain of both animals and humans, of relevance to sex differences in behavior and mental health.

The current study focused on investigating in depth the sub-regions of the limbic brain that have previously been shown to express *Cyp19a1* mRNA. Furthermore, it sets the stage for future studies on the role of cell type-specific *Cyp19a1* expression in different brain regions, and of *Cyp19a1* in neurons and astrocytes. As the analysis of the co-expression experiments is very time consuming, this study concentrated on markers for glutamatergic and GABAergic neurons as well as for *Slc1a3,* since there has been evidence for expression of *Cyp19a1* in these cell types [9, 39, 40, 46]. The experiments with the additional markers in one animal per sex are to be seen as a pilot study, giving an insight into, rather than evidence for, which other cell types might express *Cyp19a1*. Thus, more in-depth, hypothesis-free, characterization of cells expressing *Cyp19a1* through single-cell sequencing is encouraged, as well as the investigation of other regions, such as the cerebral cortex, the brainstem, the cerebellum, and the thalamus, for which there is evidence of *Cyp19a1* expression [10, 11]. Such investigations may be relevant to clinical phenotypes as recently pointed by a study on aromatase and nicotine in the human thalamus [37].

Owing to age-dependent *Cyp19a1* expression, with the highest expression prenatally when aromatase plays an important role in the sexual differentiation of the brain [23], the rats in the current study were killed at PNW 10 which corresponds to young adulthood in humans. This ensured that the animals were sexually mature while not having undergone reproductive senescence transition yet, as many sex differences in numerous neuropsychiatric disorders appear concomitantly with a hormonal transition (e.g., puberty, menopause). Previous studies examining *Cyp19a1* expression have focused on a similar time period [10–12], however it is plausible that sex differences could vary throughout the lifespan. There is a need to investigate these hormonal transition phases with regard to sex differences in *Cyp19a1* expression in the brain to gain a better understanding of the underpinnings of sex differences in mental health.

## Conclusions

The present study mapped and characterized the distribution of *Cyp19a1*^+^ cells in the limbic brain of young adult rodents. The MeP and the BNST were the regions with the highest percentage of *Cyp19a1*^+^ cells, while sex differences were widespread. Within the BNST, MPA, VMH; MeP and AHi, a higher percentage of *Cyp19a1*^+^ cells was detected in males compared to females. GABAergic and glutamatergic co-expression was found in a region-specific manner. While in most regions the *Cyp19a1*^+^ cells were identified as GABAergic, within the cortical-like regions of the amygdala the majority of *Cyp19a1*^+^ cells were glutamatergic. *Cyp19a1* expression in *Slc1a3*^+^ astrocytes was mainly present in the BNST, MPR and cortical-like regions of the amygdala. Few and non-significant sex differences were detected for the identity of *Cyp19a1*^+^ cells. The findings add to the literature and set the ground for studies on the behavioral implications of sex-specific and region-dependent expression of aromatase in the limbic brain, which might be linked to sex differences in mental health.

### Supplementary Information

**Additional file 1: Table S1.** Riboprobes used for FISH experiments. **Table S2. **Results of the co-expression experiments with additional markers.

## Data Availability

The datasets used and/or analyzed during the current study are available from the corresponding author on reasonable request.
