# Supplementary material for: Sex differences in distribution and identity of aromatase gene expressing cells in the young adult rat brain
Source: Biol Sex Differ. 2023 Sep 1;14:54. doi: 10.1186/s13293-023-00541-8 (PMC10474706; doi:10.1186/s13293-023-00541-8)
Supplement: Supplementary file 1 — Additional file 1: Table S1. Riboprobes used for FISH experiments. Table S2. Results of the co-expression experiments with additional markers. [file 13293_2023_541_MOESM1_ESM.docx]

Additional file 1

Table S1. Riboprobes used for FISH

| RIBOPROBES | | | | | | |
| --- | --- | --- | --- | --- | --- | --- |
| mRNA | ref sequence | riboprobe | | homology with rat sequence | | |
|  |  | sequence | size | homology | ref sequence | sequence |
| *mCyp19a1* | NM_007810.4 | 536-1474 | 938 bp | 92% | NM_017085.2 | 806-1742 |
| *rGad1* | NM_017007.2 | 189-728 | 539 bp |  |  |  |
| *mGad2* | NM_008078.2 | 397-1162 | 765 bp | 94% | NM_012563.2 | 134-897 |
| *rSlc17a7* | NM_053859.3 | 128-635 | 507 bp |  |  |  |
| *mSlc17a6* | NM_080853.3 | 2314-3244 | 930 bp | 92% | NM_053427.1 | 1971-2904 |
| *mSlc1a3* | NM_148938.3 | 1349-2007 | 658 bp | 96% | NM_019225.2 | 1044-1702 |
| *rChat* | NM_001170593.1 | 524-1064 | 540 bp |  |  |  |
| *mGfap* | NM_010277.3 | 379-1334 | 955 bp | 95% | NM_017009.2 | 300-1255 |
| *mAif1* | NM_001361501.1 | 212-845 | 633 bp | 94% | NM_017196.3 | 69-567 |
| *mPvalb* | NM_001330686.1 | 92-962 | 870 bp | 95% | NM_022499.2 | 12-538 |
| *mCck* | NM_001284508.2 | 306-788 | 482 bp | 92% | NM_012829.2 | 324-634 |
| *mSst* | NM_009215.1 | 142-547 | 405 bp | 97% | NM_012659.2 | 192-579 |
| *rTh* | NM_012740.4 | 456-1453 | 997 bp |  |  |  |
| *rSlc18a2* | NM_013031.2 | 704-1444 | 740 bp |  |  |  |

Table S2. Results of the co-expression experiments with additional markers.

|  | **sex** | **Acb** | **AHi** | **MeP** | **MPAA** | **PCo** | **STIA** | **BNST** | **VMH** |
| --- | --- | --- | --- | --- | --- | --- | --- | --- | --- |
| ***Chat*** | m | 0 | 0 | 0 | 0 | 0 | 0 | 0 | 0 |
|  | f | 0 | 0 | 0 | 0 | 0 | 0 | 0 | 0 |
| ***Th*** | m | 0 | 0 | 0 | 0 | 0 | 0 | 0 | 0 |
|  | f | 0 | 0 | 0 | 0 | 0 | 0 | 0 | 0 |
| ***Slc18a2*** | m | 0 | 0 | 0 | 0 | 0 | 0 | 0 | 0 |
|  | f |  | 0 | 0 | 0 | 0 | 0 | 1 | 1 |
| ***Cck*** | m | 2 | 10 | 9 | 2 | 3 | 5 | 3 | 5 |
|  | f | 2 | 5 | 3 | 6 | 10 | 6 | 0 | 2 |
| ***Sst*** | m | 5 | 3 | 3 | 5 | 1 | 13 | 2 | 1 |
|  | f | 4 | 13 | 5 | 0 | 4 | 9 | 9 | 10 |
| ***Pvalb*** | m | 0 | 0 | 0 | 0 | 0 | 0 | 0 | 0 |
|  | f | 0 | 0 | 0 | 0 | 4 | 0 | 0 | 0 |
| ***Gfap*** | m |  | 3 | 1 | 1 | 6 | 0 | 1 | 0 |
|  | f | 1 | 0 | 7 | 2 | 8 | 7 | 2 | 3 |
| ***Aif1*** | m | 0 | 0 | 0 | 0 | 3 | 0 | 1 | 2 |
|  | f |  | 6 | 15 | 4 | 4 | 2 | 5 | 0 |

The results shown in this table are based on one rat from each sex and presented as the percentage of *Cyp19a1*^+^/cell marker^+^ nuclei from all *Cyp19a1*^+^ nuclei in a region. Abbreviations: Acb: nucleus accumbens; AHi: amygdalo-hippocampal nucleus; BNST: bed nucleus of the stria terminalis; f: female; m: male; MeP: posterior part of the medial amygdaloid nucleus; MPR: medial preoptic region; PCo: posterior part of the cortical amygdaloid nucleus; VMH: ventromedial hypothalamic nucleus.
